# Parental Hesitancy and Attitude Concerning COVID-19 Vaccine and Its Side Effects in Saudi Arabia, Eastern Region

**DOI:** 10.7759/cureus.48776

**Published:** 2023-11-14

**Authors:** Rabab A Majzoub, Omalbneen H Alrofaie, Lena K Almotreb, Sarah K Alateeq, Fidaa R Bin obaid

**Affiliations:** 1 Pediatrics, King Faisal University, Alahsa, SAU; 2 Medicine and Surgery, Prince Saud Bin Jalawi Hospital, Hufof, SAU; 3 Medicine and Surgery, King Faisal University, Hofuf, SAU; 4 Family Medicine, Eastern Health Cluster, Dammam, SAU; 5 Medicine and Surgery, King Faisal University, Hufof, SAU

**Keywords:** sar- cov-2, covid-19 vaccine, parents’ attitude, vaccine side effects, covid 19

## Abstract

Background

Vaccine hesitancy poses a substantial challenge to the field of public health. There are various factors that influence the willingness of parents to vaccinate their children. Addressing the factors contributing to vaccine hesitancy within the community has the potential to facilitate the development of more effective approaches for global vaccination initiatives. This study aims to assess parents' perspectives regarding the immunization of children aged five to 12 against COVID-19, including their experiences with adverse effects, as well as the factors that influence their hesitancy or acceptance of the vaccine in the Eastern Province of Saudi Arabia.

Methods

A web-based, cross-sectional study utilized an independently administered online questionnaire. The validated questionnaire was distributed to study participants through social networking platforms in order to recruit individuals from various locations in the eastern region of Saudi Arabia, such as Dammam, Al-Hassa, Al-Jubail, Ras Tanura, Dhahran, Al-Khobar, and Al-Qatif.

Results

The study encompassed a total of 399 participants. The most commonly reported adverse effects among the first and second children were pain at the injection site (1st child: 267 (66.70%), second child: 263 (66.20%)) and fever (1st child: 171 (43.10%), second child: 187 (47.50%)). A significant proportion of the participants, specifically 139 individuals (35%), expressed apprehension regarding immunization. This concern stemmed from various factors, including the fear of experiencing adverse effects, skepticism regarding the vaccine's efficacy, and exposure to potentially harmful information about the vaccine. Ninety-nine participants, accounting for 25% of the sample, indicated their agreement with the safety of COVID-19. Additionally, 104 participants, constituting 26% of the sample, held the belief that receiving vaccination aids in the prevention of severe illnesses caused by COVID-19. Notably, the most prevalent reason for vaccine hesitancy among participants was the fear of experiencing adverse effects. A total of 132 individuals, accounting for 33% of the participants, identified healthcare providers, including physicians and scientists, as reliable and trustworthy sources of vaccine information. A statistically significant correlation was observed between the demographic variables of the participants and their acceptance of the vaccine.

Conclusion

The study observed an acceptable vaccination rate among children aged five to 12 for the COVID-19 vaccine. Based on the investigation results, the primary apprehension expressed by parents regarding the immunization of their offspring pertained to the potential negative consequences associated with the vaccine. Nonetheless, it was observed that adverse effects were reported in less than fifty percent of vaccinated children. Addressing the concerns pertaining to the COVID-19 vaccination can enhance global participation in the immunization program aimed at mitigating future pandemics.

## Introduction

The World Health Organization (WHO) labeled the coronavirus disease (COVID-19) outbreak a pandemic in March 2020. The severe acute respiratory syndrome coronavirus 2 (SARS-CoV-2) virus now causes COVID-19, an infectious disease that affects people of all ages and causes respiratory illness [1].

Decades of research and evolutionary advances in immunology, vaccinology, and adjuvant biology have resulted in the quick development and clinical testing of viable vaccines to prevent the SARS-CoV-2 pandemic. The Centers for Disease Control and Prevention (CDC) Advisory Committee on Immunization Practices (ACIP) recommended BNT162b2 (Pfizer-BioNTech) mRNA COVID-19 vaccine for persons 12 -15 years of age (this report refers to them as adolescents) on May 12, 2021, and for children aged five to 11 on the second of November 2021-2 [2-4]. In clinical trials with children and adolescents, the vaccine's effectiveness was better than 90% [5].

As with other more common vaccines, side effects are inevitable and may be unpredicted [6]. According to a survey carried out in the United States on the side effects of Pfizer-BioNTech COVID-19 vaccination that was given to children aged five to 11 years, 86.2% of all study participants experienced at least one local reaction at the site of injection, and 66.6% experienced at least one systemic reaction of mild to moderate severity [6]. Fatigue, headaches, chills, and new or worsening muscle pain were the most prevalent complaints [6]. The findings are consistent with the safety findings from the Pfizer-BioNTech COVID-19 vaccine prior approval trials, which were delivered to five to 11-year-old children [3].

Vaccinations for children under the age of 18 are frequently decided by their parents [7]. According to the findings of a web-based survey developed by the study's researchers in partnership with vaccine specialists from the Department of Public Health in England, 48% of parents or guardians would favor COVID-19 vaccine for their children under the age of 18 [7]. According to a Chinese study, parental approval of COVID-19 vaccination was much lower among physicians and nurses (44.5%) than among factory workers (72.6%) [8]. One research conducted in Saudi Arabia on the adverse effects of the COVID-19 Pfizer-BioNTech vaccination in children aged 12 to 18 years found that the most prevalent side effect was soreness or redness at the injection site [9].

Another study that was carried out in Saudi Arabia to examine the prevalence and associated causes of vaccination hesitancy in children during the COVID-19 era found that most mothers firmly agreed with the need for vaccination, with the main reason being concerns about side effects [10].

Concerningly, parental vaccine reluctance in the Kingdom of Saudi Arabia is likely to impact their children's immunization status [11]. In a research done in Saudi Arabia to pinpoint what influences parental acceptance and rejection, showed that immunization is the best strategy to prevent COVID-19 infection in themselves and their families and to stop the disease from spreading among the general population [12]. Furthermore, parents disclosed that they refused to vaccinate both themselves and their children because of the efficiency (18.7%) and adverse effects (22.9%) of the COVID-19 vaccine [11,12]. Additionally, some research shows a connection between participants' sociodemographic details and their perceptions of the effectiveness of the vaccine during the pandemic [11]. A study done in various Eastern Mediterranean Region (EMR) countries to assess the link between parents' socio-demographic variables and the status of COVID-19 vaccination of their children revealed that parents' age, education level, occupation, prior COVID-19 infection, and vaccination status all had a significant influence on their children's immunization rates [13]. Interestingly, parents who work or study in the health field are less likely to vaccinate their children than parents who work in other fields [13].

Most studies performed in Saudi Arabia focused on parents’ willingness and intentions regarding getting their child immunized against COVID-19 to begin with [14]. Our current study aims to investigate the COVID-19 vaccine's common side effects in children aged five to 12, assess parental attitudes, assess determinants of parental hesitancy, and determine the association between demographic characteristics and vaccination hesitancy in Saudi Arabia's eastern province. To the best of our knowledge, this study is the first in the literature to point out the determinants of parental hesitancy toward vaccination among those who have already received the vaccine in the region.

## Materials and methods

Study design

A cross-sectional study was performed on children between the ages of five and 12 utilizing a self-administered online questionnaire for reporting adverse effects of the COVID-19 vaccine and parental acceptance towards immunization of children at this age against COVID-19. The study was conducted in Eastern province regions of Saudi Arabia including Dammam, Al-Hassa, Al-Jubail, Ras Tanura, Dhahran, Al-Khobar, and Al-Qatif during the period 2022 to 2023.

Study population and sample size

The study was successfully carried out in Saudi Arabia's Eastern Province, with a sample size of 384 participants. The sample size of parents of children between five to 12 years old is 384, which was obtained using the Richard Geiger equation, with a margin of error of 5%, a confidence of 95%, and a response distribution of 50% for the total children's population aged five to 12 (taken from the National Demographic Statistics Year 2016 participants) [15]. The information was collected randomly using an electronic questionnaire. After giving informed consent, participants filled out the questionnaire on Google Forms to be involved in the study. 

Selection criteria

The study encompassed parents residing in the Eastern province with children aged five to 12 years, regardless of their COVID-19 vaccination status. The study excluded parents residing outside of the Eastern province or whose children were less than five or older than 12 years of age. 

Data collection

The primary method of data collection was survey-based and the sampling technique used was simple random. The survey was developed in Arabic using Google Forms, and the link was shared across social media platforms as well as through quick response (QR) code in various community gathering places in the Eastern Province regions of Saudi Arabia, such as shopping, and family parks from 2022 to 2023. The questionnaire was created following an extensive literature search that included PubMed, Medline, Google Scholar, and other databases [9,16], with the goal of identifying potential and frequent short-term adverse effects of the COVID-19 vaccine and discouraging parental approval or denial of children's COVID-19 vaccination. The first section contained an introduction to the study's objectives, contact information for the study authors to allow communication among research authors and participants, and a consent part where participants agreed to participate in the research. The second section gathered general data regarding the participants in the research (parents of children), such as gender, age, academic degree, marital status, and number of children. The third section gathered general data regarding the children, including sex, age, and long-term conditions, whether the child has been immunized against COVID-19 and the number of doses received, the adverse effects associated with the COVID-19 vaccination, and the timing the persistence of the adverse effects. The fourth and fifth sections were designed to collect information about the COVID-19 vaccine's adverse effects and parental attitudes toward COVID-19 and its vaccine (Appendix 1).

Validation and reliability testing of the questionnaire

A pilot investigation was carried out to determine the study's feasibility, reliability, and validity. The reliability of the constructs was tested using Cronbach’s alpha, and all the alpha values were above 0.7. Validity was determined subjectively using face validity (expert opinions) and objectively by discriminate validity using the Fronell-Larcker criterion, and the square roots of average variance extracted (AVE) for each construct were found to be higher than the correlation between the same construct and the other constructs, which is a sign of discriminate validity (all values of AVE were more than 0.5).

Ethical issues

The confidentiality of the patient and the privacy of their data are priorities. Nothing that could raise ethical concerns, such as the participants' identities, was used. The ethical approval was granted by King Faisal University's College of Medicine's ethical committee (KFU-REC-2022-NOV-ETHICS301).

Statistical analysis

The data were analyzed using SPSS (BM Corp. Released 2019. IBM SPSS Statistics for Windows, Version 26.0. Armonk, NY: IBM Corp). The Chi-square test was used to test the association. Frequencies were used to present categorical variables while mean and standard deviation were used to represent continuous variables. A p-value of less than 0.05 for a 95% confidence interval was considered significant.

## Results

Our study included 399 participants in total. The majority of the informants (82% were mothers), and around 39% of the participants ages ranged between 31 and 40 years. Regarding the education level of participants, more than half (57% had a bachelor's degree), 52% were employed, and 22% worked in the educational field. The vast majority of the participants (94% were married), and the rest were either divorced or widowed. More than half of the participants (51.6%) earned less than 15 thousand Saudi Arabian Riyals (SAR) as their monthly income. Regarding the number of children among the participants, less than one quarter, (22% had three children). Moreover, 16.3% of respondents reported their children had chronic diseases, distributed as asthma (6.5%), DM (6.3%), 5.3% sickle cell anemia (5.3%), other diseases (3%), and leukemia (2%). Overall, about half of the participants (48.9% of their five to 12-year-old children) had received the vaccine against COVID-19. Of those who had vaccinated their children, less than one quarter (22.6% of their first child) had received two doses, and 19.0% had received one dose of the vaccine against COVID-19 (Table 1).

**Table 1:** Demographic characteristics of participants and COVID-19 vaccination (n = 399).

Demographic Variables	Value or Category	Frequency (Percent %)
Informant:	Mother	327 (82.0%)
	Father	72 (18.0%)
Age:	20-30 years	108 (27.1%)
	31-40 years	158 (39.6%)
	41-50 years	102 (25.6%)
	51-60 years	31 (7.8%)
Education level:	Intermediate school	14 (3.5%)
	High school	56 (14.0%)
	Bachelor's degree	230 (57.6%)
	Diploma	48 (12.0%)
	Postgraduate	51 (12.8%)
Employment status:	Employed	210 (52.6%)
	Retired	30 (7.5%)
	Unemployed	159 (39.8%)
Working field:	Security field	11 (2.8%)
	Health field	62 (15.5%)
	Education field	88 (22.1%)
	Engineering field	11 (2.8%)
	Business field	27 (6.8%)
	Craftsmanship	3 (0.8%)
	Freelance work	17 (4.3%)
	Other	11 (2.8%)
	Not working at all	169 (42.4%)
Marital status:	Married	375 (94.0%)
	Single	24 (6.0%)
Family monthly income:	More than 15000 SAR	193 (48.4%)
	Less than 15000 SAR	206 (51.6%)
Number of children:	One	58 (14.6%)
	Two	87 (21.8%)
	Three	90 (22.6%)
	More than three	164 (41.1%)
Do any of your children (ages 5-21) suffer from any chronic diseases?	No	334 (83.7%)
	Yes, diabetes	25 (6.3%)
	Yes, asthma	26 (6.5%)
	Yes, sickle cell anemia	22 (5.5%)
	Yes, leukemia	8 (2.0%)
	Yes, Others	12 (3.0%)
Did all children (ages 5-12) receive the vaccine against COVID-19?	Yes	195 (48.9%)
	No	204 (51.1%)
Number of first-child doses of the vaccine against COVID-19 (if you answered the previous this question with yes) you:	Not applicable	204 (51.1%)
	One dose	76 (19.0%)
	Two doses	90 (22.6%)
	Three doses	27 (6.8%)
	Four doses	2 (0.5%)

**Figure 1:**
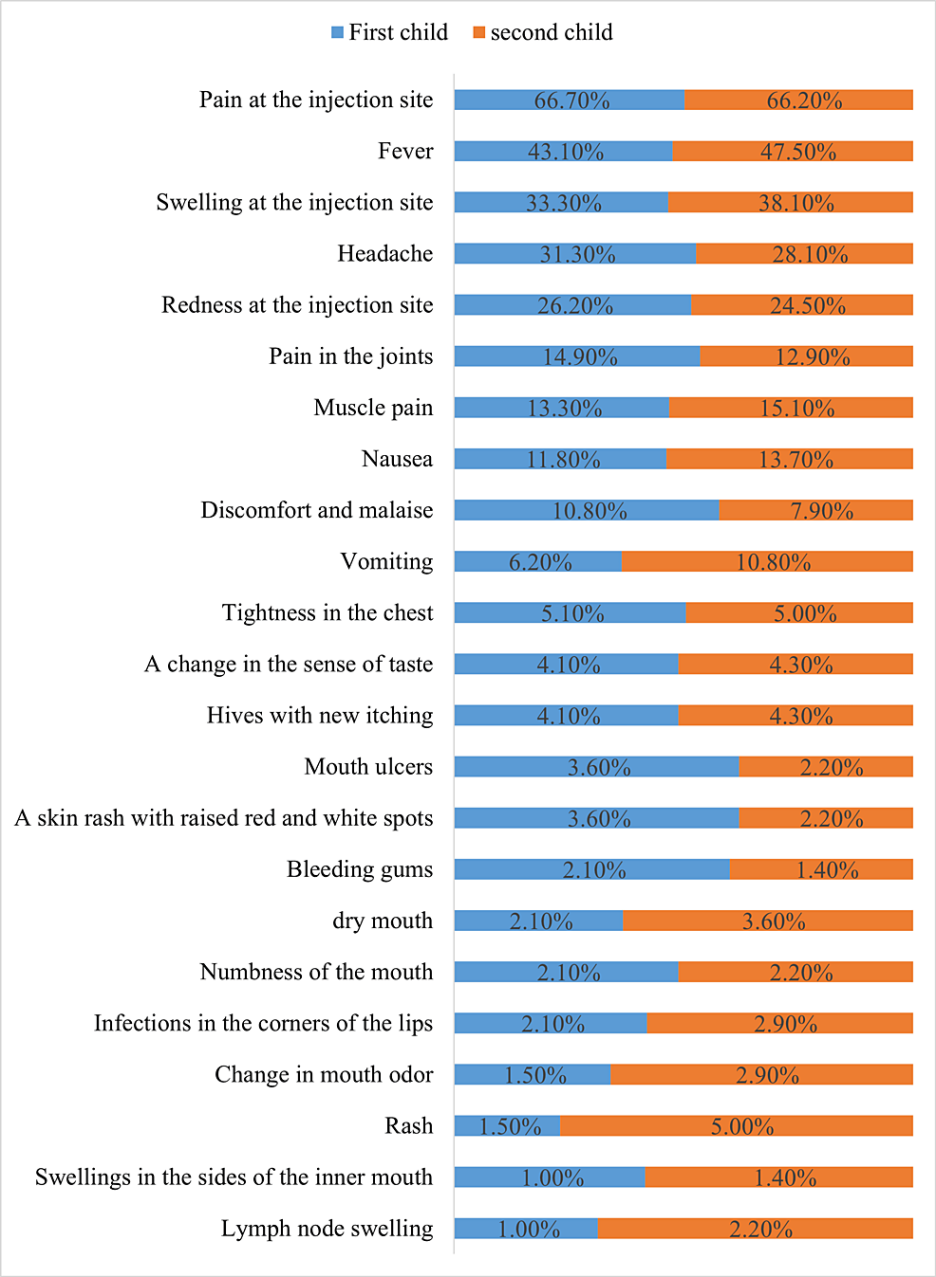
Side effects of COVID-19 vaccine

Regarding the side effects of the COVID-19 vaccine, less than half of respondents (48%) reported that their children had side effects. Also, more than one-third (35.1%) reported that the symptoms appeared after the first day of the vaccine, and one-quarter (25%) mentioned that the symptoms persisted for one to three days. The most common side effects experienced by the first and second children were pain at the injection site (first child: 66.70%, second child: 66.20%), fever (first child: 43.10%, second child: 47.50%), swelling at the injection site (first child: 33.30%, second child: 38.10%), and headache (first child: 31.30%, second child: 28.10%). (Table 2, Figure 1).

**Table 2:** Side effects of COVID-19 vaccine.

Side effects of COVID-19 vaccine	Value or Category	Frequency (Percent)
Are the side effects that appeared on your children similar to each other?	Yes	71 (17.8%)
	No	123 (30.8%)
	Don’t have side effect	205 (51.4%)
When did the symptoms of your child/children begin after taking the vaccine?	After the first day	140 (35.1%)
	After the second day	42 (10.5%)
	After more than 3 days	13 (3.3%)
	Symptoms not appeared	204 (51.1%)
What is the average duration of side effects for your child?	1-3 days	101 (25.3%)
	3-5 days	50 (12.5%)
	5-7 days	32 (8.0%)
	One week – 4 weeks	9 (2.3%)
	More than 1 month	3 (0.8%)
	Symptoms not appeared	204 (51.1%)

Regarding the attitude about COVID-19, 42% of respondents thought it was extremely likely that COVID-19 is a serious disease; 45% believed it was somewhat likely that they and their children are susceptible to COVID-19, and one quarter expressed their agreement with the safety of the COVID-19 vaccine. In addition, 26% strongly agreed that getting vaccinated helps prevent severe illnesses from COVID-19. Moreover, there were many concerns during the pandemic: 71% of the respondents feared that a family member would contract the virus, 57% worried that they would become infected, and 42% expressed concern over the possibility of death (Table 3).

**Table 3:** Attitude towards COVID-19 and its vaccination (=399).

What do you think about COVID-19?	Value of category	Frequency (Percent)
I think COVID-19 is a serious disease.	Extremely likely	171 (42.9%)
	Somewhat likely	115 (28.8%)
	Somewhat unlikely	40 (10.0%)
	Extremely unlikely	29 (7.3%)
	It depends on who gets COVID-19	44 (11.0%)
I think that my child/ren and I are vulnerable to COVID-19.	Extremely likely	143 (35.8%)
	Somewhat likely	180 (45.1%)
	Somewhat unlikely	47 (11.8%)
	Extremely unlikely	29 (7.3%)
Do you think you have enough information about COVID-19 vaccines?	Extremely likely	200 (50.1%)
	Somewhat likely	117 (29.3%)
	Somewhat unlikely	56 (14.0%)
	Extremely unlikely	26 (6.5%)
How likely would you think the information about COVID-19 vaccines are reliable?	Extremely likely	140 (35.1%)
	Somewhat likely	170 (42.6%)
	Somewhat unlikely	56 (14.0%)
	Extremely unlikely	33 (8.3%)
In general, about COVID-19 vaccination vaccines are safe.	Strongly agree	96 (24.1%)
	Agree	100 (25.1%)
	Neutral	128 (32.1%)
	Disagree	47 (11.8%)
	Strongly disagree	28 (7.0%)
Vaccination against COVID-19 can help to prevent serious illnesses that occurs due to the COVID-19	Strongly agree	104 (26.1%)
	Agree	115 (28.8%)
	Neutral	107 (26.8%)
	Disagree	51 (12.8%)
	Strongly disagree	22 (5.5%)
COVID-19 vaccine could have common side effect like any other vaccine	Strongly agree	163 (40.9%)
	Agree	154 (38.6%)
	Neutral	51 (12.8%)
	Disagree	21 (5.3%)
	Strongly disagree	10 (2.5%)
If you have side effect from COVID-19 vaccine probably will disappear after a few day	Strongly agree	108 (27.1%)
	Agree	156 (39.1%)
	Neutral	78 (19.5%)
	Disagree	38 (9.5%)
	Strongly disagree	19 (4.8%)
My concerns about related side effects prevent me from taking a vaccine for the prevention of COVID-19.	Strongly agree	106 (26.6%)
	Agree	99 (24.8%)
	Neutral	92 (23.1%)
	Disagree	77 (19.3%)
	Strongly disagree	25 (6.3%)
Have you or someone you know ever had a bad reaction to a vaccine?	Yes	186 (46.6%)
	No	136 (34.1%)
	Not sure	77 (19.3%)
What are you most worried about during this COVID-19 pandemic?	Fear of becoming infected myself	230 (57.6%)
	Fear of a family member becoming infected	287 (71.9%)
	Death	169 (42.4%)
	Financial related worries	58 (14.5%)
	Job-related worries	51 (12.8%)
	Food insecurity related worries	56 (14.0%)
	Unavailability of vaccines	86 (21.6%)
	Being a plot or conspiracy	88 (22.1%)
	Being forced to take a medication	57 (14.3%)
	Being forced to take a vaccine	105 (26.3%)
	I am not worried about any issues	42 (10.5%)

Regarding the acceptance and hesitancy of the COVID-19 vaccine, more than one-third of the participants (35%) were hesitant about the vaccination due to fearing side effects, thinking the vaccine might not be effective (15.8%), having read dangerous information about the vaccine (12%), or thinking the vaccine might interact with other diseases (11%). However, 40% of their children had received a vaccine against COVID-19 because it was mandatory, and 15% because they believed the vaccine would protect their children against COVID-19 (Table 4).

**Table 4:** Causes of acceptance and hesitancy of COVID-19 vaccine (n=399).

Causes of acceptance and refusal of vaccine against COVID-19	Value of category	Frequency (Percent )
What are your concerns about getting your child vaccinated against COVID-19?	Fear about adverse effects of vaccination	142 (35.6%)
	Children have good immunity as they don't need vaccination	3 (0.8%)
	The vaccines might not be effective	63 (15.8%)
	Death of one of the family members	25 (6.3%)
	have read dangerous information about the vaccine	49 (12.3%)
	Not enough studies on the vaccine and its side effects	3 (0.8%)
	The vaccine may interact with other diseases	46 (11.5%)
	No concerns	68 (17.0%)
If your children receive a vaccine against COVID-19, choose the reason for acceptance	Because it is a mandatory	160 (40.1%)
	Protect my children against COVID-19	60 (15.0%)
	Help to end the pandemic	17 (4.3%)
	Reduce the severity of symptoms during the disease	36 (9.0%)

**Figure 2:**
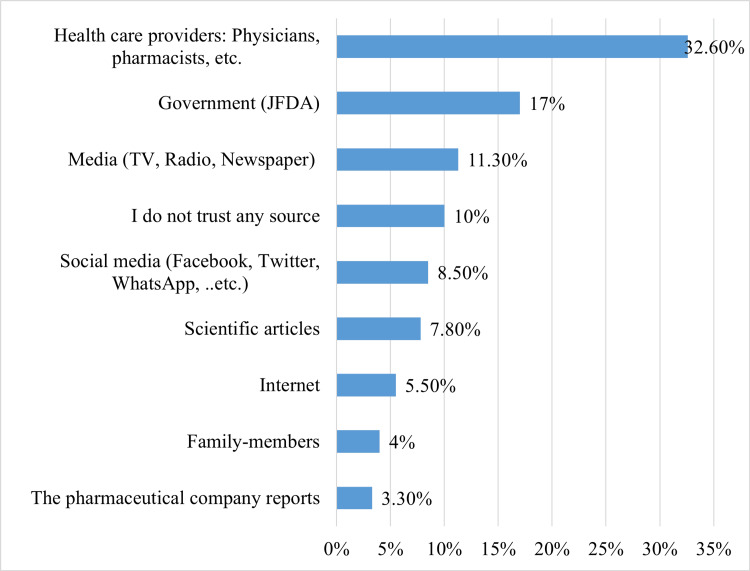
Sources of information regarding COVID-19 vaccine (n=399).

Regarding the sources of information about the COVID-19 vaccine around one-third of participants (32.6%) mentioned healthcare providers (such as physicians and pharmacists, etc.) as a trusted source for information about vaccines, followed by the government (17.0%) and the internet (11.3%). Social media and pharmaceutical company reports were trusted by 8.5% and 3.3%, respectively, while 10.0% reported not trusting any source (Figure 2).

The demographic features of the participants (working field and income) had a statistically significant link with vaccine acceptability, according to the Chi-square test (p-value= 0.011). However, several other factors were not found to be associated. Furthermore according to the Chi-square statistic, fear of side effects, the belief that the vaccine might not be effective, and having read dangerous information about the vaccine were all significantly associated with hesitance to receive the COVID-19 vaccine at the 0.05 level. (Tables 5, 6).

**Table 5:** Association of demographic characteristics and status of vaccination (n=399). * The Chi-square statistic is significant at the 0.05 level.

Variables		Did all children (ages 5-12) receive the vaccine against COVID-19?		Chi-square	p-value
		Yes	No		
Informant	Mother	48.6%	51.4%	0.045	0.833
	Father	50.0%	50.0%		
Age	20-30 years	43.5%	56.5%	3.952	0.267
	31-40 years	47.5%	52.5%		
	41-50 years	52.9%	47.1%		
	51-60 years	61.3%	38.7%		
Education	Intermediate school	64.3%	35.7%	3.760	0.439
	high school	42.9%	57.1%		
	Bachelor's degree	47.0%	53.0%		
	Diploma	54.2%	45.8%		
	Postgraduate	54.9%	45.1%		
Employment status	Employed	51.0%	49.0%	2.225	0.329
	Retired	56.7%	43.3%		
	Unemployed	44.7%	55.3%		
Working field	Security field	81.8%	18.2%	16.185	0.040*
	Health field	45.2%	54.8%		
	Education field	55.7%	44.3%		
	Engineering field	72.7%	27.3%		
	Business field	51.9%	48.1%		
	Craftsmanship	100.0%	0.0%		
	Freelance work	35.3%	64.7%		
	Other	36.4%	63.6%		
	Not working at all	43.8%	56.2%		
Marital status	Married	47.7%	52.3%	3.236	0.072
	Single	66.7%	33.3%		
Family monthly income	More than 15000	59.6%	40.4%	17.171	< 0.001*
	Less than 15000	38.8%	61.2%		
Chronic disease	No	48.5%	51.5%	.112	0.738
	Yes	50.8%	49.2%		
Number of children	Less than 5	46.9%	53.1%	1.664	0.197
	5 and above	54.2%	45.8%		

**Table 6:** Association between concerns about getting COVID-19 vaccine and status of vaccination (n=399). * The Chi-square statistic is significant at the 0.05 level

What are your concerns about getting your child vaccinated against COVID-19?	Did all children (ages 5-12) receive the vaccine against COVID-19?		Chi-square	p-value
	Yes	No		
	Column %	Column %		
No mention of any concern	23.6%	10.8%	18.16	0.011*
Fear about adverse effects of vaccination	30.3%	40.7%		
Children have good immunity as they don't need vaccination	1.0%	0.5%		
The vaccines might not be effective	11.8%	19.6%		
The vaccines may cause some health problems in the future	0.0%	0.0%		
Death of one of the family members	6.2%	6.4%		
I have read dangerous information about the vaccine	13.3%	11.3%		
There are not enough studies on the vaccine and its side effects	1.0%	0.5%		
The vaccine may interact with other diseases	12.8%	10.3%		

## Discussion

This study aims to identify Saudi parents' hesitancy and behaviors regarding COVID-19 vaccinations of their children aged five to 12 years old and to investigate the causes of acceptance and hesitancy that may encourage or prevent Saudi parents from vaccinating their children against the COVID-19 virus. The COVID-19 immunization in Saudi Arabia was expanded to children over the age of 12 in June 2021 and to children aged five to 11 in January 2022 [17].

We found that about half of the participants (48.9%), their children, aged five to 12, had received the vaccine against COVID-19. These findings were slightly higher than the findings reported in Jordan (30%) [18], Turkey (36.3%) [19], Iraq (38%) [20], Saudi Arabia (44%) [12] and England (48.2%) [7]. Our findings were less than previous studies in the United States (63%) [21], Canada (63%)[22], Japan (64.7%) [23], Egypt (65.6%) [24], Italy (68.5%)[25], China (72.6%) [8], Malaysia (73.6%) [26] and UAE (75.1%) [27].

This study demonstrates the actual percentage of children who have been vaccinated, in contrast to prior studies, which have only shown parents' intentions to vaccinate their children.

The primary concerns of parents in the current study about vaccinating their children were the possible adverse effects of the vaccine (35%), thinking the vaccine might not be effective (15.8%), having read dangerous information about the vaccine (12%), or thinking the vaccine might interact with other diseases (11%). These findings are consistent with earlier research in Egypt, where the primary worries were the vaccine's adverse effects (68.3%) and ideas of conspiracies (18%) [24], and in Malaysia, where the common reasons against vaccination were uncertainty regarding the new vaccine (96.1%), concerns regarding vaccine ingredients (93.2%), shortage of vaccine information provided from clinicians (82.3%), and the notion that vaccines are unsafe (79.8%) [25]. Several previous studies have found that parents are hesitant to vaccinate their children because of worries regarding adverse side effects: 42.9% in Saudi Arabia [14], 46.8% in Romania [28], 61.5% in Boston [29], 76.9% in Turkey [19] and 84.1% in Korea [16].

The current study revealed that the primary factors that prompted parents to give their children the COVID-19 vaccine were that it was mandatory (40%), and to protect their children against COVID-19 (15%). Moreover, working field and high income had a statistically significant correlation with vaccine acceptance. Previous studies in Malaysia showed that the reasons for willingly immunizing children were for their protection (99.4%), protection of their family members (99.3%), and effectiveness (98.2%) [26]. Protecting the child’s health was the main reason for parent’s acceptance among previous studies in Saudi Arabia (24.1%) [14], UAE (55.5%) [27] and Turkey (75.5%) [19].

According to the current study, approximately one-third of the respondents (32.6%) cited healthcare providers as a reliable source of vaccine information, followed by the government (17.0%), the internet (11.3%), and social media (8.5%). These findings are consistent with previous studies in the United States, where 64.88% of respondents relied on healthcare providers for information about the vaccine, while 15% reported relying on social media [30]. In contrast, a previous study in Palestine reported that social media, the internet, and healthcare professionals were the main sources of information about the COVID-19 vaccine (22.07%, 18.74%, and 11.92%, respectively) [31]. These variations in information sources highlight the importance of understanding the local context when developing communication strategies and policies to encourage vaccine uptake.

The current study reports that 42% of respondents thought it was extremely likely that COVID-19 is a serious disease, 45% believed it was somewhat likely that they and their children are susceptible to COVID-19, and one quarter expressed their agreement with the safety of the COVID-19 vaccine. In addition, 26% strongly agreed that getting vaccinated helps prevent severe illnesses from COVID-19. Moreover, there were many concerns during the pandemic, where 71% of the respondents feared that a family member would contract the virus, 57% worried that they would become infected, and 42% expressed concern over the possibility of death. These findings are similar to findings in previous studies in Najran, Saudi Arabia, in which nearly 70% of parents agreed that the COVID-19 vaccine was effective [32] and in Italy, where 78.7% agreed that COVID-19 is a serious disease and 42.3% agreed that it is preventable [25].

Regarding the side effects of the COVID-19 vaccine, less than half of those who responded (48%) reported that their children had side effects. More than one-third (35.1%) reported that the symptoms appeared after the first day of the vaccine, and one-quarter (25%) mentioned that the symptoms persisted for one to three days. The most frequent adverse effects experienced by the first and second children were pain at the injection site (first child: 66.70%, second child: 66.20%), fever (first child: 43.10%, second child: 47.50%), swelling at the injection site (first child: 33.30%, second child: 38.10%), and headache (first child: 31.30%, second child: 28.10%). These findings are consistent with previous studies at the national level, which reported that 50.4% of children experienced side effects. The majority of them (78.9%) said that adverse effects began one day after receiving the immunization, and 65.7% reported that they lasted one to three days. In addition, the most documented effect was pain at the injection site (15.3%) [33]. The World Health Organization (WHO) stated that COVID-19 vaccines, like other vaccines, could cause adverse effects. However, most of these adverse effects were minor and of short duration [34].

Limitations of the study

Our study demonstrated some limitations. One of them is that as there is no central immunization record in Saudi Arabia for the COVID-19 vaccine and the parents reported the child’s vaccination status, this could potentially be underestimated or overstated due to social desirability or recollection bias. In addition, instead of a direct face-to-face interview, study results were recorded via a web-based, self-administered survey. This may result in prejudice in reporting their responses.

## Conclusions

The rate of children (ages five to 12) who received the COVID-19 vaccine was acceptable in the current study. Although the study found that the main concern of parents about vaccinating their children was the possible side effects of the vaccine, the side effects were reported in less than half of the vaccinated children. The most common side effects experienced were pain at the injection site, fever, swelling at the injection site, and headache that appeared after the first day of the vaccine and lasted for a few days. Public health authorities and other organizations should provide reliable and easy-to-understand information through a variety of media, including social media platforms, to influence population attitudes towards vaccination and emerging health problems.

## Appendices

Appendix 1

Parent's willingness and attitude concerning COVID-19 vaccine and its side effects in Saudi Arabia, Eastern region questionnaire.

**Table 7:** Participant consent.

Do you agree to participate in the questionnaire for the research?	Yes
	No

Part 1

Parents information 

**Table 8:** Parents information.

Question	Options
Gender:	Female ( the mother )
	Male ( the father )
Age:	20-30
	31-40
	41-50
	51-60
Education (Degree):	Intermediate school
	High school
	Bachelor's degree
	Diploma
	Postgraduate
Employment status:	Employed
	Unemployed
	Retired
Working field:	Security field
	Health field
	Education field
	Engineering field
	Business field
	Freelance work
	Other
Marital status:	Married
	Single
Family monthly income:	More than 15000
	Less than 15000
Number of children:	Short answer

Part 2

Child information

**Table 9:** Child information.

Question	Options
Age of 1^st^ child:	5 year
	6 years
	7 years
	8 years
	9 years
	10 years
	11 years
	12 years
Age of the 2^nd^ child:	5 year
	6 years
	7 years
	8 years
	9 years
	10 years
	11 years
	12 years
Do any of your children (ages 5-21) suffer from any chronic diseases?	No
	Yes, diabetes
	Yes, asthma
	Yes, sickle cell anemia
	Yes, leukemia
Did all children (ages 5-12) receive the vaccine against COVID-19?	Yes
	No
Note: if the participant answer this question with NO will skip the 3^rd^ part and move to 4^th^ part	
Number of first-child doses of the vaccine against COVID-19 (if you answered the previous question with yes ) you:	One dose
	Two doses
	Three doses
	Four doses
Number of second child doses of the vaccine against COVID-19 (if you answered the previous question with yes)	One dose
	Two doses
	Three doses
	Four doses

Part 3

Side effects of COVID-19 vaccine

**Table 10:** Side effects of COVID-19 vaccine.

Question	Options
Are the side effects that appeared on your children similar to each other?	Yes
	No
What are the effects of the effects that your first child suffered after taking the vaccine? (you can choose more than one answer):	Pain at the injection site
	Swelling at the injection site
	Redness at the injection site
	Fever
	Headache
	Nausea
	Vomiting
	Tightness in the chest
	Pain in the joints
	Muscle pain
	Discomfort and malaise
	Lymph node swelling
	Rash
	Hives with new itching
	A skin rash with raised red and white spots above the surface of the skin
	Infections in the corners of the lips
	Mouth ulcers
	Numbness of the mouth
	A change in the sense of taste
	dry mouth
	Change in mouth odor
	Bleeding gums
	Swellings in the sides of the inner mouth
What are the effects of the effects that your first child suffered after taking the vaccine? (you can choose more than one answer):	Pain at the injection site
	Swelling at the injection site
	Redness at the injection site
	Fever
	Headache
	Nausea
	Vomiting
	Tightness in the chest
	Pain in the joints
	Muscle pain
	Discomfort and malaise
	Lymph node swelling
	Rash
	Hives with new itching
	A skin rash with raised red and white spots above the surface of the skin
	Infections in the corners of the lips
	Mouth ulcers
	Numbness of the mouth
	A change in the sense of taste
	dry mouth
	Change in mouth odor
	Bleeding gums
	Swellings in the sides of the inner mouth
When did the symptoms of your child/children begin after taking the vaccine?	After the first day
	After the second day
	After more than 3 days
What is the average duration of side effects for you child?	1-3 days
	3-5 days
	5-7 days
	One week – 4 weeks
	More than 1 month

Part 4

What do you think about COVID-19?

**Table 11:** What do you think about COVID-19?

Question	Options
I think COVID-19 is a serious disease.	Extremely likely
	Somewhat likely
	Somewhat unlikely
	Extremely unlikely
	It depends on who gets COVID-19
I think that I and my child(ren) are vulnerable to COVID-19.	Extremely likely
	Somewhat likely
	Somewhat unlikely
	Extremely unlikely
Do you think you have enough information about COVID-19 vaccines?	Extremely likely
	Somewhat likely
	Somewhat unlikely
	Extremely unlikely
How likely would you think the information about COVID-19 vaccines are reliable?	Extremely likely
	Somewhat likely
	Somewhat unlikely
	Extremely unlikely
In general, about COVID-19 vaccination vaccines are safe.	Strongly agree
	Agree
	Neutral
	Disagree
	Strongly disagree
Vaccination against COVID-19 can help to prevent serious illnesses that occurs due to the COVID-19	Strongly agree
	Agree
	Neutral
	Disagree
	strongly disagree
COVID-19 vaccine could have common side effect like any other vaccine	Strongly agree
	Agree
	Neutral
	Disagree
	Strongly disagree
If you have side effects from the COVID-19 vaccine probably it will disappear after a few day	Strongly agree
	Agree
	Neutral
	Disagree
	Strongly disagree

Part 5

What do you think about COVID-19 vaccination?

**Table 12:** What do you think about COVID-19 vaccination

Question	Options
Have you or someone you know ever had a bad reaction to a vaccine?	Yes
	No
	Not sure
What are you most worried about during this COVID-19 pandemic? (Tick not more than 5)	Fear of becoming infected myself
	Fear of a family member becoming infected
	Death
	Financial related worries
	Job-related worries
	Food insecurity related worries
	Unavailability of vaccines
	Being a plot or conspiracy
	Being forced to take a medication
	Being forced to take a vaccine
	I am not worried about any issues
	Other
Who do you trust the most for information about vaccines?	Media (TV, Radio, Newspaper)
	Internet
	Social media (Facebook, Twitter, WhatsApp, ..etc.)
	Health care providers: Physicians, pharmacists, etc.
	Family-members
	Government (JFDA)
	The pharmaceutical company reports
	Scientific articles
	I do not trust any source

Part 6

Causes of acceptance and refusal of vaccination against COVID-19

**Table 13:** Causes of acceptance and refusal of vaccination against COVID-19

Question	Options
My concerns about related side effects will prevent me from taking a vaccine for the prevention of COVID-19.	Strongly agree
	Agree
	Neutral
	Disagree
	Strongly disagree
What are your concerns about getting your child vaccinated against COVID-19? (you can choose more than one option)	Adverse events after vaccination
	The vaccines have not been sufficiently
	The vaccines might not be effective enough
	Death of one of the family members
	Others
	The vaccines may cause some health problems in the future
If your children receive a vaccine against COVID-19, choose the reason for acceptance	Because it is a mandatory
	Protect my children against COVID-19,
	Help to end the pandemic
	Reduce the severity of symptoms during the disease
